# Experiences of Left-Handed Dental Students in Australia

**DOI:** 10.15694/mep.2018.0000083.1

**Published:** 2018-04-12

**Authors:** Chris Lee, Kelsey Pateman, Ratilal Lalloo

**Affiliations:** 1 School of Dentistry

**Keywords:** improvements, dental education, left-handed dental students, perceived difficulties

## Abstract

This article was migrated. The article was marked as recommended.

**Background:** The aim of this study is to understand how left-handed (LH) dental students perceive their education and identify areas for improvement in their training.

**Methods:** All LH students in their senior clinical years of their programs, at the nine Australian dental schools, were invited to participate in the study, by completing an online questionnaire.

**Results:** A total of 40 students responded to the survey. The clinical disciplines ranked most often in the top 3 for difficulty were operative (restorative) dentistry (73%), followed by similar percentages for periodontics (49%), prosthodontics (46%), oral surgery (45%) and endodontics (44%). Areas for improvement that ranked highest were instructors and supervisors, and dental chair orientation (both at 26%). Operating tools and equipment, and dental chair orientation (68%) were most often ranked in the top 3, followed by the interior design of the room (63%) and instructors and supervisors (61%).

**Keywords:** dental education, left-handed dental students, perceived difficulties, improvements

**Conclusion:** The findings suggest that LH students perceived greater difficulty from the inconvenience caused by inadequate design of the surgery and chair, and due to a lack of personalised teaching. The findings of this study support a need for alterations to learning environment to better support LH students in learning specific procedures and increasing access to individualised teaching approaches.

## Introduction

The practice of dentistry requires mastery of a specific skillset, involving the precise execution of finely coordinated movements with the arms, hands and fingers. Developing finely tuned manual dexterity skills can be demanding for students during the formative years of clinical training. Left-handed (LH) students may face additional challenges in the availability of LH dental chairs, instruments and LH teachers and clinical supervisors ( Al-Johany, 2013; Kapoor, Puranik, & Uma, 2016).

Around 2 - 30% of the global human population are left-handed (LH),(“RIGHTLEFTRIGHWRONG - An investigation of Handedness - Some Facts, Myths, Truths, Opinions and Research,”) and there is a similar reported range of left-handedness in the dental profession ( Al-Johany, 2013; Gutwinski et al., 2011; Hardyck & Petrinovich, 1977; Kapoor et al., 2016). Historically, immense social pressure urged left-handers to work in a right-handed (RH) manner or to adapt to working with RH equipment with little or no support from training institutions ( Henderson & Stephens, 1995; Sheppard, 1995; Whitmore, 1995). Awareness of the needs of left handed clinicians has improved over time and now it is widely accepted that being LH is just a difference, rather than a limitation.

An important focus of dental education is to develop suitable dexterity for dental work ( Canakci, Tan, Orbak, & Tezel, 2002). The hand skills of an operator directly relates to the performance of the operator, and quality of care provided ( Canakci et al., 2002; Kapoor et al., 2016). Ideally, dental schools should be properly equipped and prepared to offer quality education to all its students. Many dental practices and dental schools are, however, designed and equipped for RH students ( Brown, 1995). This may compromise the performance of LH students. Most, if not all, of the dental techniques and procedures taught in dental schools are taught to fit RH students ( Brown, 1995).

Musculoskeletal health is important for all members of the dental team ( Hayes, Cockrell, & Smith, 2009). There are numerous risk factors implicated in the development of musculo-skeletal disorders (MSD) associated with dentistry ( Silva et al., 2016; Valachi & Valachi, 2003a, 2003b). Poor ergonomics can force clinicians into awkward positions known to be problematic over time. Information on the prevalence of MSD’s among students is limited, with conflicting findings ( Hayes et al., 2009). An Australian study found no significant differences in MSD symptoms reported based on handedness in a survey of general dental practitioners ( Marshall, Duncombe, Robinson, & Kilbreath, 1997). In their systematic review, Hayes et al. ( Hayes et al., 2009) noted that the risk of MSD was inversely related to years of practice, meaning that recent graduates and early career practitioners may likely experience greater MSDs than longer term practitioners. This may place LH students at increased risk of MSD, especially when newly graduated, and arguably, less in control of their working environments.

Research on handedness in dentistry is scarce. To our knowledge, there is currently no research specific to Australia. The limited research reported generally broadly argue that the education for LH students needs to be improved ( Canakci et al., 2002; Kapoor et al., 2016). There is consensus that there is a significant improvement in performance in LH dental students and professionals when working in a LH manner. Some of the research specifically highlights that LH students have problems with having RH instructors, affecting their quality of learning ( Al-Johany, 2013). Much of the research conducted is however not sufficiently detailed to specify which aspects of dental education needs to be improved. The research either focussed on only certain branches of dentistry (restorative, periodontics, and orthodontics), or focussed on the dental practice itself, not the education. The aim of this study is to understand how LH dental students in Australia perceive their education and identify areas for improvement in their training in Australian dental schools.

## Methods

This research was non-funded and was approved by The University of Queensland School of Dentistry Research Ethics Committee (Project No. 1603).

All left-handed students in their senior clinical years of their programs, at the nine Australian dental schools (The University of Queensland, Griffith University, The University of Sydney, The University of Melbourne, La Trobe University, The University of Western Australia, Charles Sturt University, James Cook University, and The University of Adelaide), were invited to participate in the study, by completing an online questionnaire. In the universities with a five-year program, students in years 3 to 5 were invited; and those with a four-year program, students in years 2 to 4 were invited. All students were invited by the respective contact person at each dental school. To maximise the response rate, three reminders were sent fortnightly to the contact person at each dental school.

Questions for the online survey (Checkbox® Survey - https://www.checkbox.com/) were extracted from research conducted by Al-Johany ( Al-Johany, 2013), the questions were adapted and modified to suit the local environment and the aim of the study. Additional general questions regarding the degree of handedness and social pressure were used from an online hand preference questionnaire developed by the University of Indiana ( Holder, 2001). The questionnaire contained 25 questions in total, and was divided into three sections: social, equipment, and education. The social section related to social pressure for being LH, for example, assessing discrimination in the participants’ personal life and university education. The equipment and education sections assessed dental schools’ preparation and equipment for LH students. The equipment section specifically assessed the “hardware” of dental schools, such as the dental chair, design of working areas, and instrumentation. The education section related to the teaching and training aspects of the course, for example, assessment, guidance, instructors, performance and difficulties. Most questions in the survey were close-ended: the participants were expected to answer to what degree or frequency related to the questions, except for two questions. For the two questions, the list of options regarding the branches or disciplines of dentistry that LH students experience the most difficulty with and improvements required in dental education for LH students were provided. The respondents were asked to number the options from most to least difficult and most to least important respectively. Open-ended questions were also provided, to provide participants with opportunities to elaborate on difficulties experienced and any suggestions for improvements in dental education, and the survey itself.

The responses were downloaded from the online survey tool as an Excel file and converted to SPSS for analysis. Frequency distribution were analysed and reported for the close-ended responses, due to the nature of the data and number of respondents. The responses for the ranking of disciplines and areas for improvements were reported graphically. Inductive content analysis was used to analyse open ended responses ( Hsieh & Shannon, 2005). The inductive data analysis involved revision of all responses to identify key issues, followed by formation of a coding framework, and applied to the full data set. The initial coding and categorisation was performed independently by two authors (KP and CL). Rigour of data analysis was ensured through transparency of coding and through discussion of the coding process and outcomes among the research team. Disagreements and discrepancies in data coding were resolved through discussion.

## Results

A total of 40 responses were available for the analysis. Of these almost 60% were male, and the mean age was 24 years. The age ranged from 20 to 35 years with 68% younger than 25 years. There was an even distribution of responses across the three clinical years of study.

Just more than a half (53%) reported being fully LH while the others could do some things with their right hand (ambidextrous). Almost two-thirds (65%) felt it was not difficult to be LH. More than third (35%) were never forced or suggested to change their handed by others; however, 43% were sometimes encouraged to change. Specifically in their dental program, 45% were never suggested while 23% reported to sometimes receiving this advice.

Almost all respondents (90%) felt their clinical performance was better when they worked in a LH manner, and 70% reported they sometimes worked in a RH manner. 40% reported experiencing problems sometimes doing dental work. Almost none felt their patients were uncomfortable being treated by a LH person, 73% however felt their patients sometimes noticed they were LH.

**Figure 1.**
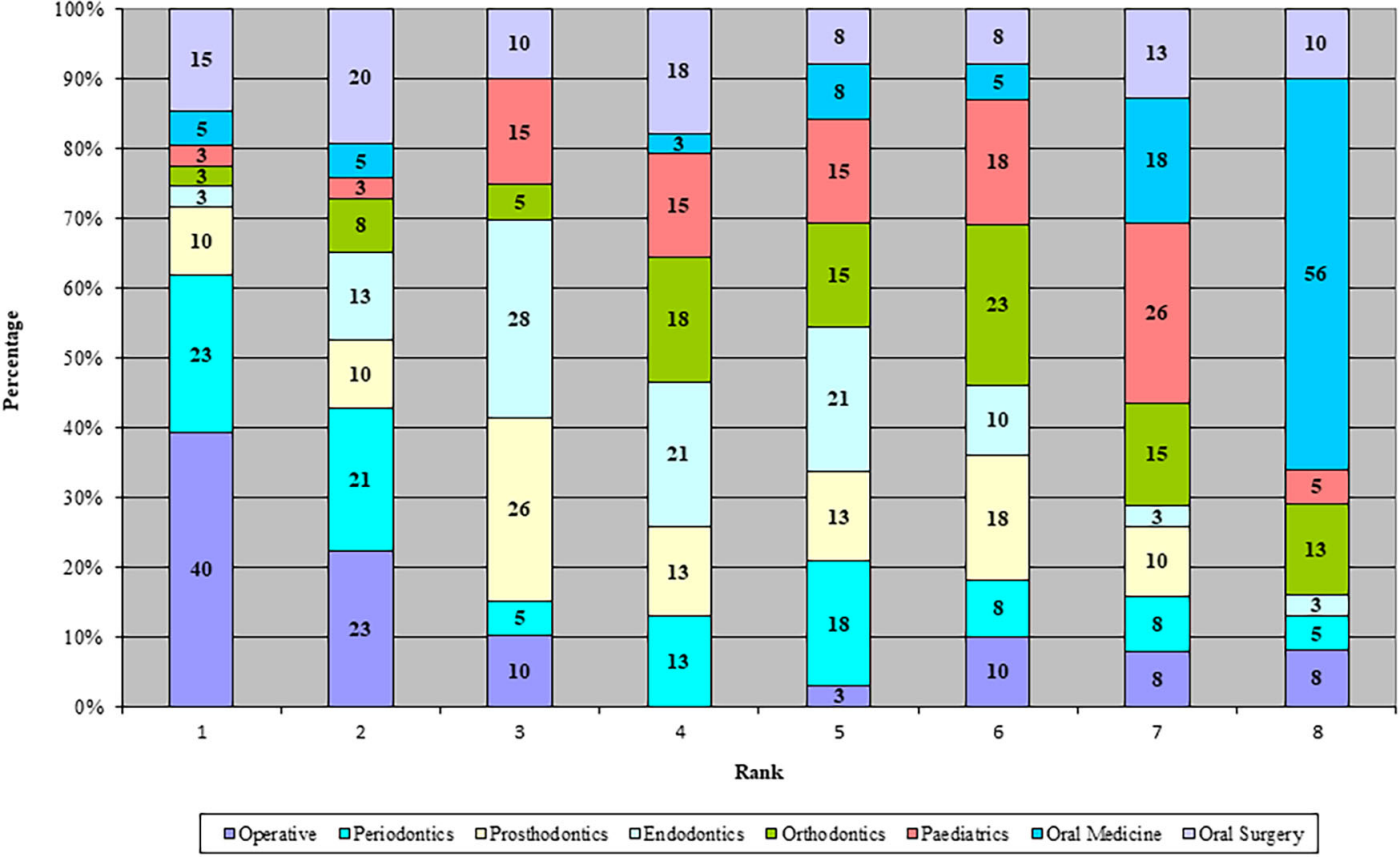
Ranking of clinical disciplines by difficulty - 1 being most difficult to 8 being least difficult

When asked to rank clinical disciplines in terms of difficulty being LH, operative (restorative) dentistry was most often ranked in the top 3 (73%), followed by similar percentages for periodontics (49%), prosthodontics (46%), oral surgery (45%) and endodontic (44%) ( Figure 1). The disciplines least often ranked in the top 3 were oral medicine (13%), orthodontics (15%) and paediatrics (21%).

Thirty participants responded to the open-ended question, elaborating on difficulties experienced with specific disciplines. Responses identified difficulties with positioning, both with space limitations posed by surgery design, and also difficulty adapting RH instructions to the LH equivalent. Operator comfort and visibility was compromised during operative procedures by limitations in the chair design (i.e. short handpiece cords, set-up and order of handpieces, position of suction) or surgery design (orientation of the clean/dirty bench).

Difficulty with the periodontics discipline was caused by needing to independently learn the left-handed versions of core techniques, such as establishing the correct seating arrangements, patient positioning and instrument adaptation. This may be due to a lack of supervisor knowledge to teach LH techniques.

A smaller number of open-ended responses (total n=8) also identified some situations where being able to switch between both hands was advantageous, such as in paediatrics when needing to manage an uncooperative child, administering LA, or choosing to use RH for procedures requiring strength, and LH for procedures requiring fine detail.

When asked if they had problems with RH instructors there was an even distribution across the possible options: yes (30%); maybe (33%) and no (37%). Half reported having instructors who were LH and almost none reported preferential access to them. Almost all preferred using a dental station designed for a LH person and a half felt their school was properly equipped for a LH person. When asked if they felt disadvantaged in their performance in practical assessment, again there was a spread across the possible options: yes (25%); maybe (30%) and no (45%). Almost two-thirds (63%) felt their dental assistant experienced problems assisting a LH person. Again, almost two-thirds (65%) did not know if LH dentists experienced more musculoskeletal and other medical complications compared to RH dentists. About a third felt they needed to indicate they LH in their CV and 45% felt being LH would affect them getting a job.

When asked to rank areas for improvement instructors and supervisors, and dental chair orientation were most often ranked first (both at 26%). Operating tools and equipment, and dental chair orientation (68%) were most often ranked in the top 3, followed by the interior design of the room (63%) and instructors and supervisors (61%). Areas seldom ranked in the top 3 were social pressure or acceptance (26%) and assessments (21%).

Working environment was an issue identified throughout the open-ended responses. Responses identified that having a chair that could convert to a LH set-up also needed to be positioned in a room with draws, cupboards and working benches “flipped” to a LH set-up. This was to enable increased comfort and improved ergonomics for both the clinician and the dental assistant. Responses identified that greater education was needed for clinic instructors to teach skills in both a LH and RH version, or to hire more LH instructors.

A smaller number of open-ended responses detailed feelings of discrimination and stigma or pressure associated with being a LH student. This was evident in anecdotes of comments made by supervisors about LH students being a “bloody lefty” or “irritating and a nuisance”. Some respondents also perceived that being LH would disadvantage them in seeking employment post-graduation. This led to a decision to train to work in RH chairs to improve their employability. Description of difficulties attributed to being LH were balanced by a smaller number of positive statements, including feeling well-supported by the university (n=2 responses) and that the survey validated the LH experience (n=5 responses).

## Discussion

This was the first study reporting experiences of Australian LH dental students; and contributes to a modest number of studies in the area globally. Our findings support previous studies that highlight the influence of the learning environment on the LH student experience. ( Al-Johany, 2013) The respondents of this study found operative, periodontics, and oral surgery the most difficult in descending order, and oral medicine the least difficult. LH students generally found periodontics difficult due to a variety of reasons, but there was consensus that the difficulty came not from the difficulty of the procedure itself, but more from the poor ergonomics and positioning that was not well demonstrated or taught when students were in their earlier clinical years. Open-ended comments provided further context outlining a perceived lack of expertise and availability of clinical demonstrators able to adapt teaching to LH methods. Many LH dental students were left to adapt procedures independently, and this is likely to contribute to a sense of dissatisfaction with the learning environment, and perpetuate negative associations with being LH.

The majority of respondents in the present study indicated their clinical performance was superior when working in a LH manner; yet a high proportion reported switching to work in a RH manner at times. This is likely attributable to the proportion of respondents who identified as being ambidextrous. Open-ended responses identified clinical scenarios where being able to use either left or right hands was advantageous. This indicates switching between hands may also be a deliberate or strategic decision, rather than solely a necessary adaptation to overcome the working environment.

There was some evidence that further supported that being LH was socially accepted. General public, or the patients, noticed the operators were LH, yet they had no problem with LH students regarding the operators’ preferred hand. Moreover, when asked for the parts of dental education that needs the most improvement, LH students responded that the social pressure or acceptance needed the least improvement. However, this acceptability was not reflected in attitudes towards employment. Although more than half of LH students did not feel the need to indicate their preferred hand in their CV, they still felt that being LH would affect their employment. Some LH students have commented that they were suggested from their peers or instructors that working in RH manner may increase the chance of getting employed, as catering for LH dentistry in working environment was poorer than the school environment.

There were also some concerns that LH students expressed, especially with their assistants. Dentistry is a team-based profession, yet no previous research was available regarding the relationship between LH dentistry and assisting. The students have pointed out that the assistants either did not have much experience with the LH operators ^2^, or experienced inconvenience which stemmed from lack of space and inadequate chair and surgery design. Implementing systems to train assistants to be able to work with LH students, and have separate chair and surgery equipped for LH operators, are important strategies to minimise this problem.

The variation observed in open-ended responses repositioned the difficulties associated with being left-handed within a broader frame: that for some, being left-handed was not necessarily a significant problem, with recognition that difficulties could be adapted to. This highlighted individual variation within comments, and supports the need for individualised education approaches. This may however be difficult in a highly organised environment such as a dental surgery.

While the response of 40 may be small, we estimate that this is about half of all LH dental students in Australia. This is based on the student number of about 1900 across the clinical years in the nine dental schools and an assumed LH proportion of 5%. A response rate of 50% is considered acceptable for a voluntary online survey. The study did not include a RH comparison group to assess if the experiences of LH students are different to RH students, or to students in related disciplines.

**Figure 2.**
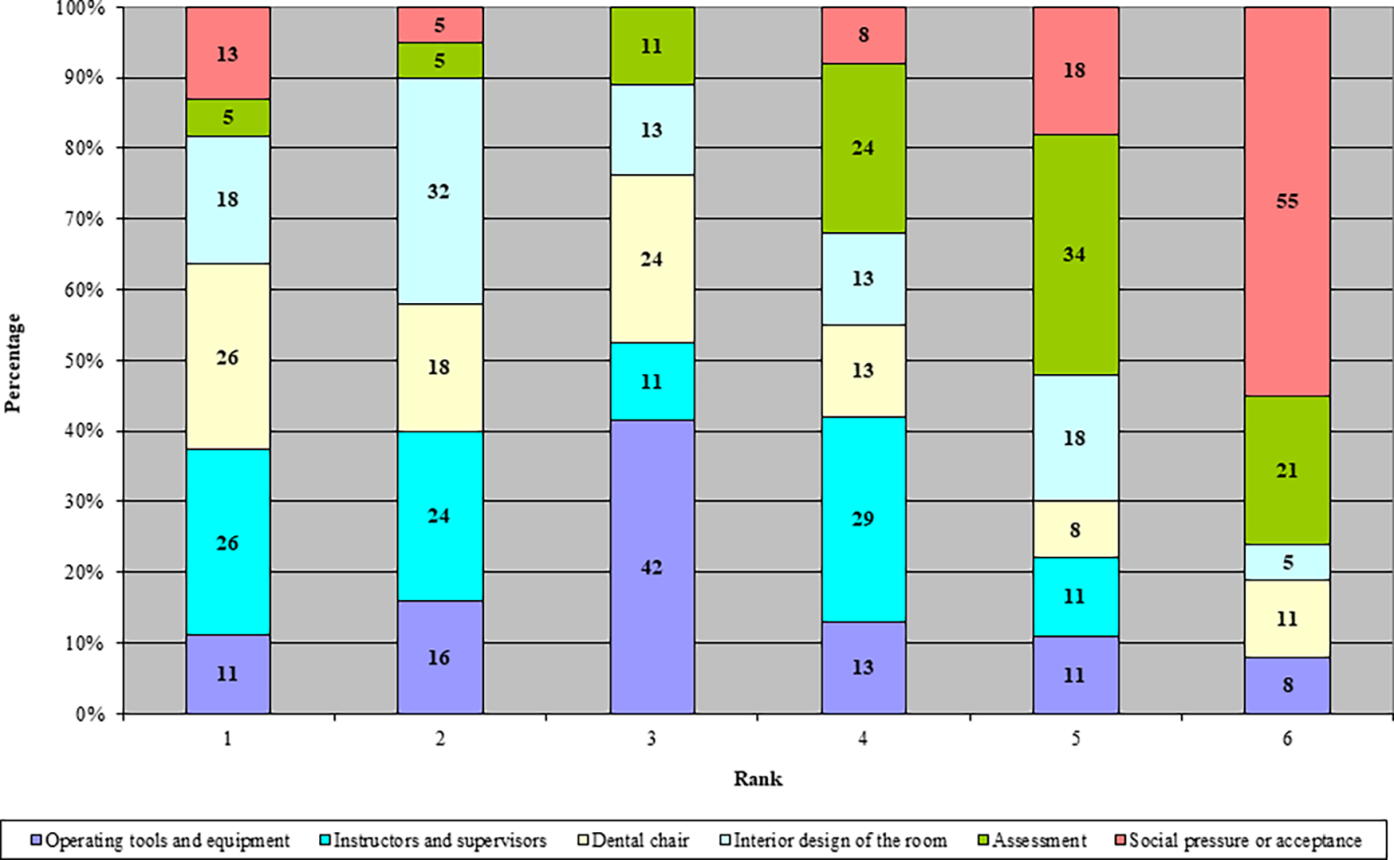
Ranking of areas for improvement - 1 being most important to 6 being least important

## Take Home Messages

• To our knowledge this was the first study reporting experiences of Australian left-handed dental students; and contributes to a modest number of studies in the area globally.
• Left-handed dental students perceive greater difficulty from the inconvenience caused by inadequate design of the surgery and chair, and also due to a lack of personalised teaching.
• There is a need for alterations to learning environment and increasing access to individualised teaching approaches.

## Notes On Contributors

Chris Lee conducted this research when he was a final year dental student in the School of Dentistry, The University of Queensland, Brisbane, Australia. He is now a general dental practitioner in Rockhampton, Queensland, Australia.

Kelsey Pateman was a PhD candidate in the School of Dentistry, The University of Queensland, Brisbane, Australia. She is a Consultant Oral Health TherapistOffice of the Chief Dental Officer, Clinical Excellence Division Department of Health, Queenland Health, Brisbane, Australia.

Ratilal Lalloo is Discipline Lead in Dental Public Health in the School of Dentistry, The University of Queensland, Brisbane, Australia.

## References

[ref1] Al-Johany S. S. (2013). A survey of left-handed dental students and interns in Saudi Arabia. J Dent Educ. 77(1),105–112. 23314474

[ref2] Brown J. M. (1995). Left handed GDPs and students. Br Dent J. 178(12),448. 10.1038/sj.bdj.4808800 7605719

[ref3] Canakci V. Tan U. Orbak R. & Tezel A. (2002). Right- and left-handed dentists in periodontal therapy. Int J Neurosci. 112(1),1–14. 12152401

[ref4] Gutwinski S. Loscher A. Mahler L. Kalbitzer J. Heinz A. & Bermpohl F. (2011). Understanding left-handedness. Dtsch Arztebl Int. 108(50),849–853. 10.3238/arztebl.2011.0849 22259638 PMC3258574

[ref5] Hardyck C. & Petrinovich L. F. (1977). Left-handedness. Psychol Bull. 84(3),385–404. 10.1037/0033-2909.84.3.385 859955

[ref6] Hayes M. Cockrell D. & Smith D. R. (2009). A systematic review of musculoskeletal disorders among dental professionals. Int J Dent Hyg. 7(3),159–165. 10.1111/j.1601-5037.2009.00395.x 19659711

[ref7] Henderson N. J. & Stephens C. D. (1995). Left handed GDPs. Br Dent J. 179(1),8. 10.1038/sj.bdj.4808814 7626337

[ref8] Holder M. (2001). Hand Preference Questionnaire. Retrieved from http://www.indiana.edu/~primate/forms/hand.html

[ref9] Hsieh H. F. & Shannon S. E. (2005). Three approaches to qualitative content analysis. Qual Health Res. 15(9),1277–1288. 10.1177/1049732305276687 16204405

[ref10] Kapoor S. Puranik M. P. & Uma S. R. (2016). Practice Perspectives of Left-Handed Clinical Dental Students in India. J Clin Diagn Res. 10(10),ZC79–ZC83. 10.7860/JCDR/2016/17550.8664 PMC5121811 27891465

[ref11] Marshall E. D. Duncombe L. M. Robinson R. Q. & Kilbreath S. L. (1997). Musculoskeletal symptoms in New South Wales dentists. Aust Dent J. 42(4),240–246. 10.1111/j.1834-7819.1997.tb00128.x 9316311

[ref12] RIGHTLEFTRIGHWRONG - An investigation of Handedness - Some Facts, Myths, Truths, Opinions and Research. Retrieved from http://www.rightleftrightwrong.com/statistics.html

[ref13] Sheppard P. N. (1995). Left handed GDPs. Br Dent J. 179(1),8. 10.1038/sj.bdj.4808815 7626338

[ref14] Silva E. Cruz I. Costa I. Lima K. Souza G. Fuscella M. & Andrade F. (2016). Left-Handed Students and Clinical Practice in Dentistry: Adaptations, Difficulties and Realities Experienced in the Academic Environment. Open Journal of Preventive Medicine. 6,247–259. 10.4236/ojpm.2016.611023

[ref15] Valachi B. & Valachi K . (2003a). Mechanisms leading to musculoskeletal disorders in dentistry. J Am Dent Assoc. 134(10),1344–1350. 10.14219/jada.archive.2003.0048 14620013

[ref16] Valachi B. & Valachi K . (2003b). Preventing musculoskeletal disorders in clinical dentistry: strategies to address the mechanisms leading to musculoskeletal disorders. J Am Dent Assoc. 134(12),1604–1612. 14719757 10.14219/jada.archive.2003.0106

[ref17] Whitmore J. (1995). Left handed GDPs. Br Dent J. 179(1),8. 10.1038/sj.bdj.4808816 7626339

