# Supplementary material for: Experiences of Left-Handed Dental Students in Australia
Source: MedEdPublish (2016). 2018 Apr 12;7:83. [Version 1] doi: 10.15694/mep.2018.0000083.1 (PMC10711996; doi:10.15694/mep.2018.0000083.1)
Supplement: Peer review history

# Reviewer report: Reviewer response for version 1

**DOI:** 10.21956/mep.19551.r28974

**Published:** 2019-03-19

**Authors:** Trevor Gibbs

**Affiliations:** 1 AMEE

This review has been migrated. The reviewer awarded 4 stars out of 5 An interesting paper that adds to the not very large number of papers written on the subject. I feel that the paper however added not a great deal to what we already know or perceive and I hope that this group of authors can now convert their work into the "so what" arena and produce some practical solutions on how to take this work forward

Reviewer Expertise:

NA

No decision status is available

# Reviewer report: Reviewer response for version 1

**DOI:** 10.21956/mep.19551.r28975

**Published:** 2018-05-16

**Authors:** P Ravi Shankar

**Affiliations:** 1 American International Medical University

This review has been migrated. The reviewer awarded 4 stars out of 5 Thank you for this interesting study. As a left hander myself, the findings of this study were of immense personal interest to me. I agree with the authors that though the situation is changing there is still prejudice against left-handers. When I was young most left-handers were forced to switch to using the ‘right’ hand by their parents and teachers. The learning and the working environment has been designed with right handers in mind which create challenges for individuals who are left-handed. The challenges of being left-handed in medicine, dentistry and other health professions are an important area of study. Left handers face a variety of challenges in their life with activities of daily living. This is a well-written and presented study. The authors have mentioned the limitations of their study. The authors could present the questionnaire they had used in the Appendix. I would also be interested in the free-text comments from the respondents.

Reviewer Expertise:

NA

No decision status is available
